# ClC-3 Chloride Channel Proteins Regulate the Cell Cycle by Up-regulating cyclin D1-CDK4/6 through Suppressing p21/p27 Expression in Nasopharyngeal Carcinoma Cells

**DOI:** 10.1038/srep30276

**Published:** 2016-07-25

**Authors:** Dong Ye, Hai Luo, Zhouyi Lai, Lili Zou, Linyan Zhu, Jianwen Mao, Tim Jacob, Wencai Ye, Liwei Wang, Lixin Chen

**Affiliations:** 1 Department of Physiology, Guangdong Pharmaceutical University, Guangzhou, China; 2 Department of Pharmacology, Medical College, Jinan University, Guangzhou, China; 3 Department of Physiology, Medical College, Jinan University, Guangzhou, China; 4 Cardiff School of Biosciences, Cardiff University, Cardiff, UK; 5 College of Pharmacy, Jinan University, Guangzhou, China

## Abstract

It was shown in this study that knockdown of ClC-3 expression by ClC-3 siRNA prevented the activation of hypotonicity-induced chloride currents, and arrested cells at the G0/G1 phase in nasopharyngeal carcinoma CNE-2Z cells. Reconstitution of ClC-3 expression with ClC-3 expression plasmids could rescue the cells from the cell cycle arrest caused by ClC-3 siRNA treatments. Transfection of cells with ClC-3 siRNA decreased the expression of cyclin D1, cyclin dependent kinase 4 and 6, and increased the expression of cyclin dependent kinase inhibitors (CDKIs), p21 and p27. Pretreatments of cells with p21 and p27 siRNAs depleted the inhibitory effects of ClC-3 siRNA on the expression of CDK4 and CDK6, but not on that of cyclin D1, indicating the requirement of p21 and p27 for the inhibitory effects of ClC-3 siRNA on CDK4 and CDK6 expression. ClC-3 siRNA inhibited cells to progress from the G1 phase to the S phase, but pretreatments of cells with p21 and p27 siRNAs abolished the inhibitory effects of ClC-3 siRNA on the cell cycle progress. Our data suggest that ClC-3 may regulate cell cycle transition between G0/G1 and S phases by up-regulation of the expression of CDK4 and CDK6 through suppression of p21 and p27 expression.

Chloride channels have been demonstrated to be the key factor in regulation of the cell cycle and cell proliferation^1^,^2^,^3^,^4^,^5^. Inhibition of chloride channels suppresses the progress of the cell cycle. Chloride channels can be classified into six categories, including the ClC superfamily of voltage-gated chloride channels^6^. ClC-3, a member of the ClC superfamily is widely expressed and hypothesized as a volume-sensitive Cl^−^ channel although debates exist^4^,^7^,^8^,^9^,^10^,^11^. Recently, the ClC-3 channel is thought to act as more than just a Cl^−^ channel^12^,^13^,^14^,^15^,^16^,^17^,^18^,^19^. Overexpression of ClC-3 chloride channel proteins has been found in many tumors including glioma and lung, liver, cervical and breast cancer^4^,^20^. The expression and distribution of ClC-3 chloride channel proteins are cell cycle-dependent^21^. These data suggest that ClC-3 may be involved in cell cycle regulation and related to occurrence of cancer cells.

The progression of cells through the cell cycle is regulated by different cyclin/CDK complexes. These molecules form the regulatory (cyclins) and catalytic (cyclin-dependent kinases, CDKs) subunits of cell cycle-regulated kinases. Cyclins can regulate the cell cycle progression by activating CDKs^22^. Cyclin D1 is a key cell cycle protein which forms a complex with CDK4 or CDK6 and plays a very important role in the G1 phase. Activity of the cyclin D1–CDK4/CDK6 complex is required to promote the progress of cells from the G0/G1 phase to the S phase. Inhibition of cyclin D1 can arrest cells at the G0/G1 phase.

The activities of cyclin/CDK complexes can be inhibited by cyclin-dependent kinase inhibitors (CDKIs), which are activated to prevent disorder in the cell cycle machinery. The CDKIs, p21 (WAF1/Cip1) and p27 (Kip1), can bind to cyclin/CDK complexes and regulate the G1–S transition by inhibition of the complex activity. Threshold kinase activity of CDKs is a crucial determinant of the cell cycle progression, and thus, inhibition of CDK activity directly or indirectly by up-regulating CDKI expression represents a rational approach to intervene with the uncontrolled proliferation of cancer cells^23^.

Evidence presented previously by us and others suggests that ClC-3 chloride channels may be involved in the regulation of the cell cycle^4^,^5^,^11^,^17^,^18^,^21^, but the underlying mechanism is not clear. It has been demonstrated by us that ClC-3 plays important roles in the activation of volume-activated and acid-activated chloride currents^4^,^11^,^19^,^21^. Interaction between ClC-3 and cyclin D1 exists, and cyclin D1 may regulate the functional activities and/or the expression of the ClC-3 chloride channel in the CNE-2Z cell (a poorly differentiated human nasopharyngeal carcinoma cell line)^24^. These data suggest that ClC-3 may regulate the cell cycle through modulation of the expression of the cyclin D1-CDKs (4, 6)-CDKIs signaling pathway.

The aim of this study was to investigate the roles of ClC-3 chloride channels in the regulation of the cell cycle and the relationship between ClC-3 chloride channels and cell cycle regulators in nasopharyngeal carcinoma CNE-2Z cells. The effects of knockdown of ClC-3 expression on the progress of the cell cycle and the expression of cyclin D1, CDK4/CDK6 and p21/p27 were observed. The requirement of p21 and p27 for the inhibitory action of ClC-3 siRNA on the cell cycle was investigated.

## Results

### ClC-3 siRNA knocked down expression of ClC-3 chloride channel proteins

**Figure 1:**
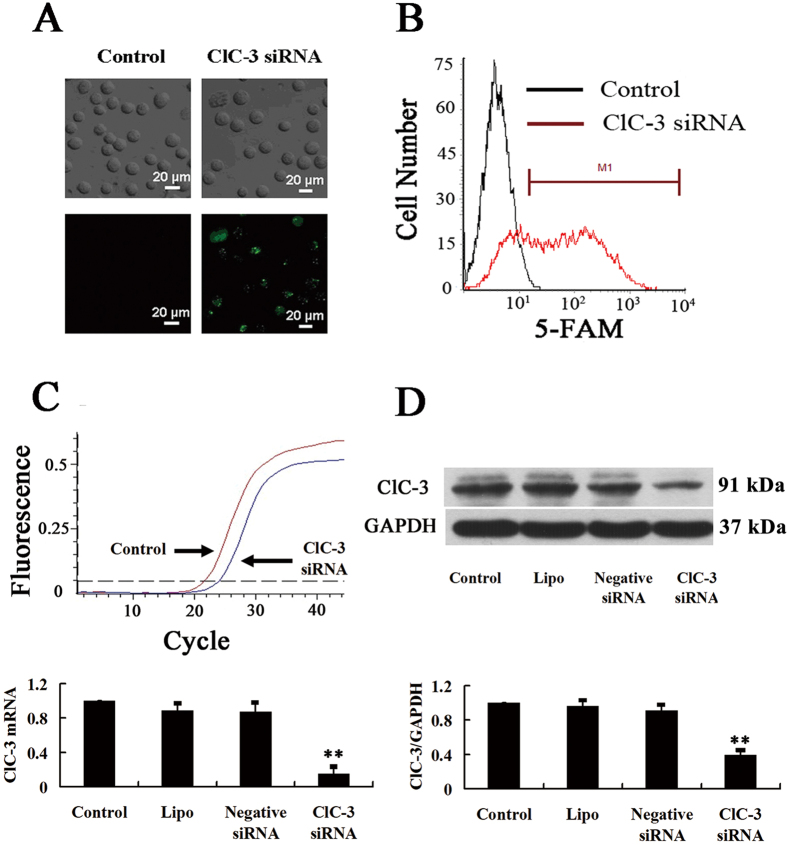
Knockdown of the expression of ClC-3 chloride channels by ClC-3 siRNA in CNE-2Z cells. (**A**) The cells were transfected with ClC-3 siRNA labelled with 5-FAM (green) for 8 h. Green fluorescence was remarkably detected in the ClC-3 siRNA- treated cells, suggesting that the siRNA had been successfully transfected into the cells. (**B**) Analysis of 5-FAM fluorescence by flow cytometry. M1 stands for the cells successfully transfected with the 5-FAM-labelled ClC-3 siRNA. (**C**) Analysis of the expression of ClC-3 mRNA in control cells and the cells treated with ClC-3 siRNA for 48 h. The upper panel shows the representative real time RT-PCR curves. The lower panel shows the quantitative analysis of ClC-3 mRNA expression. (**D**) Analysis of the expression of ClC-3 proteins and the control GAPDH proteins. The upper and lower panels show the representative blotting and the quantitative analysis, respectively. GAPDH was chosen as the reference because it is a house keeping gene and expressed steadily in many kinds of cells including nasopharyngeal carcinoma cells. Data in C and D are mean ± S.E. (n = 3). **P < 0.01 (vs. Control).

In this study, the siRNA technology was used to inhibit specifically the expression of ClC-3 chloride channel proteins. To detect the transfection efficiency, ClC-3 siRNA was labeled with 5-FAM (green) and the fluorescence was monitored with a fluorescence microscope and a flow cytometer. As shown in Fig. 1A, fluorescence could be detected in most of the cells treated with 100 nM 5-FAM-labeled ClC-3 siRNA 8 h after transfection, indicating that ClC-3 siRNA had been successfully transfected into the cells. The transfection efficiency was (88.7 ± 4.3)% (n = 3, *P* < 0.01, vs control), which was obtained by analyzing the fluorescence intensity of individual cells using flow cytometry (Fig. 1B).

To determine the efficiency of ClC-3 knockdown, we used the real time RT-PCR and Western blot techniques to detect the expression of the ClC-3 mRNA and protein. The results of the real time PCR showed that the nasopharyngeal carcinoma CNE-2Z cells expressed ClC-3 and the expression level of ClC-3 mRNA was decreased by (84.7 ± 7.2)% after 48 h treatment with ClC-3 siRNA (n = 3, *P* < 0.01, vs control, Fig. 1C). The expression of ClC-3 proteins was demonstrated by the results of the Western blotting (Fig. 1D). 48 h treatment with ClC-3 siRNA knocked down the expression of ClC-3 proteins by (60.9 ± 4.0)% (n = 3, *P* < 0.01, vs control; Fig. 1D). The treatment with the scrambled siRNA (negative control siRNA) or with the transfection reagent lipofectamine^TM^ 2000 alone did not significantly alter the expression of the ClC-3 mRNA and protein (n = 3, *P* > 0.05, vs control, Fig. 1C,D).

### ClC-3 siRNA reduced the volume-activated Cl- current and the capacity of RVD in CNE-2Z cells

**Figure 2:**
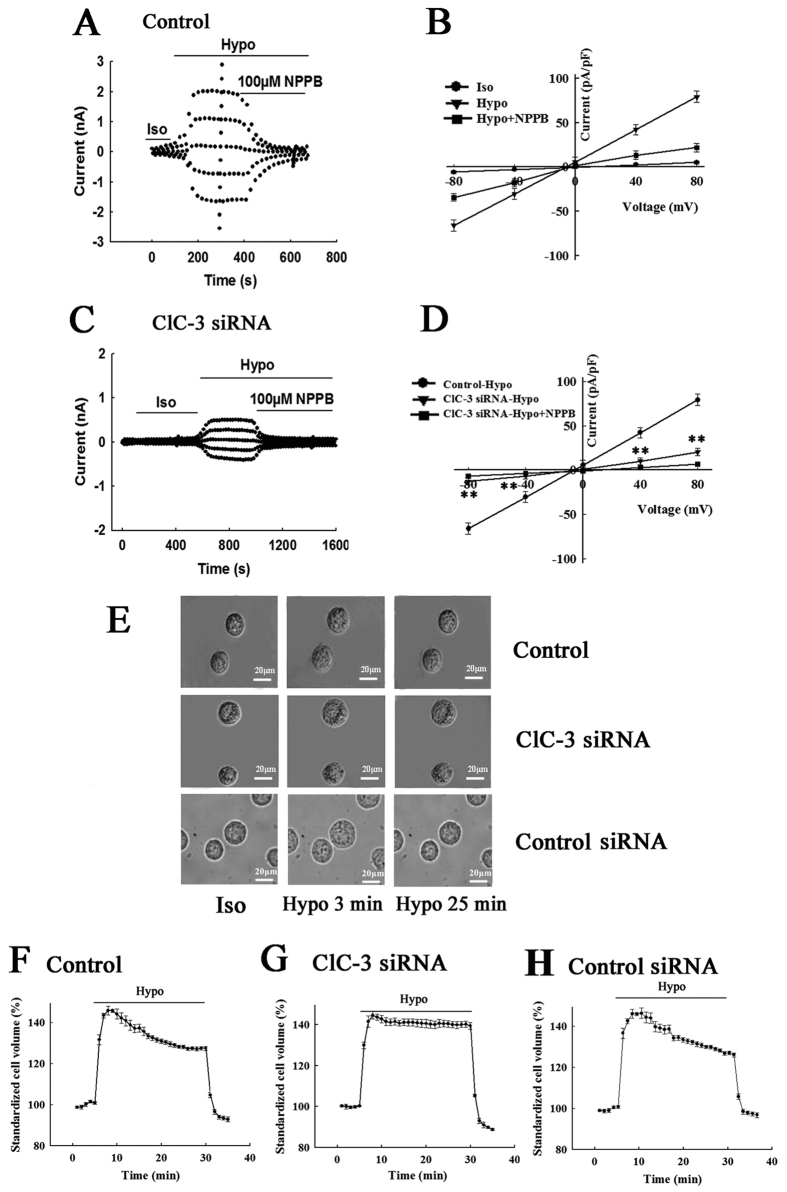
Inhibition of hypotonicity-activated Cl^−^ currents and hypotonicity-induced regulatory volume decrease (RVD) by ClC-3 siRNA in CNE-2Z cells. The typical time courses of activation of the Cl^−^ current induced by the 47% hypotonic challenge and inhibition of the current by the chloride channel blocker NPPB in the control cells and the cells transfected with ClC-3-siRNA for 48 h are shown in (**A,C**), respectively. (**B,D**) present the current-voltage relationships in the control and ClC-3 siRNA-treated groups. The voltage was held at 0 mV and stepped to ±80, ±40, 0 mV repeatedly. **P < 0.01 (vs Control-Hypo). (**E**) Images of the control cells and the cells transfected with ClC-3-siRNA or control siRNA for 48 h, taken in the isotonic solution (Iso) and 47% hypotonic solution (3 and 25 min). (F,G,H) Hypotonicity-induced cell volume changes in the control, ClC-3 siRNA-transfected and control siRNA-transfected cells (mean ± S.E., 20 and 23 cells in three experiments).

Whole-cell chloride currents at 0, ±40, ±80 mV were recorded using the patch clamp technique. As shown in Fig. 2A,B, the exposure of cells to the 47% hypotonic solution activated a large chloride current, with an average density of 74.2 ± 6.5 pA/pF at + 80 mV in control cells. The current could be inhibited by the chloride channel blocker NPPB (100 μM). In the cells treated with ClC-3 siRNA for 48 h, the hypotonicity-activated chloride current was significantly decreased to 20.58 ± 1.43 pA/pF at + 80 mV (n = 5, *P* < 0.01, vs control), and the current was inhibited by 100 μM NPPB (Fig. 2C,D). In the cells treated with the control siRNA for 48 h, the hypotonicity-activated chloride current was not significantly different from that recorded in the control cells.

The capacity of regulatory volume decrease (RVD) induced by hypotonic challenges was detected in the control and ClC-3 siRNA-treated cells. The cells were perfused in sequence with the isotonic bath solution for 5 min, hypotonic bath solution for 25 min, and the isotonic bath solution for 5 min. In the control cells, the exposure to the 47% hypotonic bath solution swelled the cells and induced a process of regulatory volume decrease. Cell volume was recovered by (41.8 ± 2.8)% in 25 min when the cells were still bathed in the hypotonic solution (20 cells in 3 experiments, *P* < 0.01, Fig. 2E,F). In the cells treated with ClC-3 siRNA for 48 h, similar cell swelling was observed, but the hypotonicity-induced RVD was decreased to (12.5 ± 4.8)% (23 cells in 3 experiments, *P* < 0.01, vs control, Fig. 2E,G). The treatments with the control siRNA did not significantly alter the hypotonicity-activated responses, compared with the control group (Fig. 2E,H).

### ClC-3 siRNA arrested CNE-2Z cells at the G0/G1 phase

**Figure 3:**
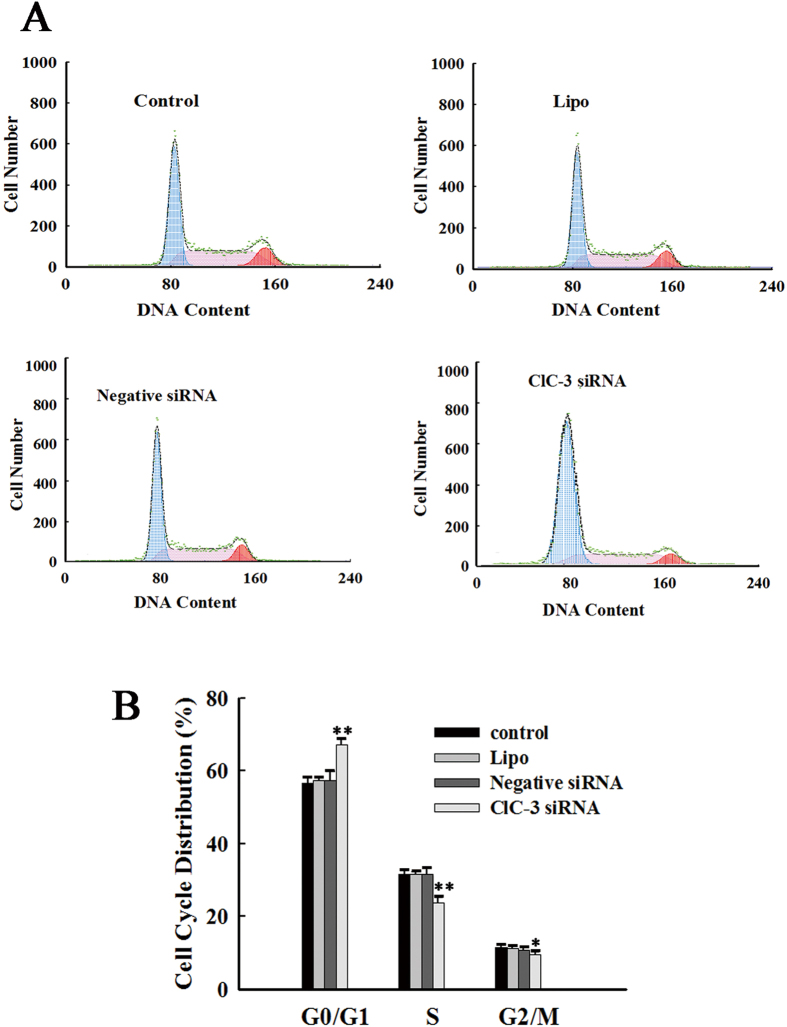
Arrest of CNE-2Z cells at the G0/G1 phase by ClC-3 siRNA. (**A**) Cell cycle distribution detected by the flow cytometry in the control cells and the cells treated with lipofectamine 2000 alone (Lipo), or with 100 nM negative siRNA or ClC-3 siRNA plus lipofectamine 2000 for 48 h. (**B**) Quantitative analysis of cell cycle distribution (mean ± S.E. of three experiments). *P < 0.05; **P < 0.01 (vs. Control).

To investigate the role of intrinsic ClC-3 in regulation of the cell cycle, the effects of knockdown of ClC-3 expression by ClC-3 siRNA on cell cycle progression were tested. Cell cycle distribution was analyzed by the flow cytometry. The results shown in Fig. 3 indicated that knockdown of ClC-3 expression inhibited the progression of cells into the S phase from the G1 phase. In the control group, (56.8 ± 2.8)% (n = 5) of the cells were in the G0/G1 phase (Fig. 3A,B). In cells treated with 100 nM ClC-3 siRNA for 48 h, the cell population in the G0/G1 phase was increased to (69.9 ± 3.0)% (n = 5, *P* < 0.01, vs control, Fig. 3A,B). The results also showed that the population of cells in S and the G_2_/M phase was decreased when the expression of the ClC-3 chloride channel protein was knocked down by the specific ClC-3 siRNA (Fig. 3). The cells in S phase was decreased from (32.1 ± 1.7)% (control, n = 5) to (23.5 ± 1.5)% (ClC-3 siRNA, n = 5, *P* < 0.01). The cells in G_2_/M phase was decreased from (11.0 ± 1.1)% (control, n = 5) to (6.5 ± 1.5)% (ClC-3 siRNA, n = 5, *P* < 0.05). The negative control siRNA and the transfection reagent lipofectamine^TM^ 2000 alone did not present any significant effects on the distribution of the cell cycle. (vs control, Fig. 3B).

### Reconstitution of ClC-3 expression rescued CNE-2Z cells from ClC-3 siRNA-induced arrest of the cell cycle

**Figure 4:**
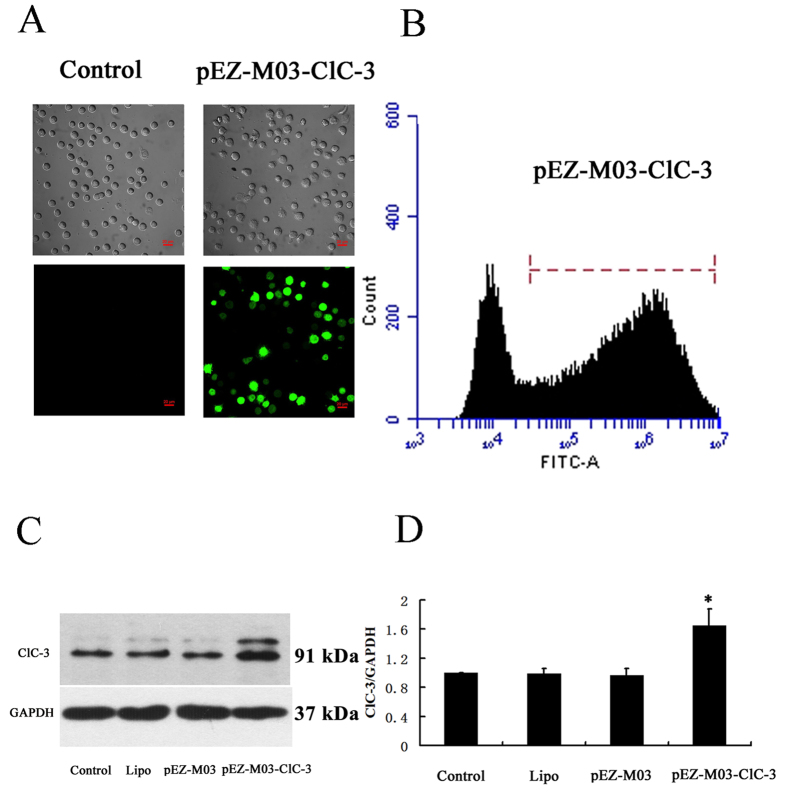
Enhancement of ClC-3 expression by constructed plasmids carrying the ClC-3 gene (pEZ-M03-ClC-3) in CNE-2Z cells. The constructed (pEZ-M03-ClC-3) and control (pEZ-M03) plasmids with GFP tag were transfected into CNE-2Z cells in the presence of Lipofectamine 2000 (Lipo). (**A**) The upper and lower panels present respectively the transmitted light and fluorescent images of the control cells and the cells transfected with pEZ-M03-ClC-3 (green) for 24 h. (**B**) Analysis of transfection efficiency by flow cytometry. The bar stands for the cells successfully transfected with pEZ-M03-ClC-3. (**C**) Representative Western blot of ClC-3 and control GAPDH proteins. (**D**) Quantitative analysis of Western blot 48 h after transfection (mean ± S.E., n = 3). *P < 0.05 (vs. Control).

The plasmid technology was used to reconstitute ClC-3 expression in this study. The transfection and expression efficiency of the constructed plasmids was first tested in the control CNE-2Z cells. The results showed that the constructed plasmid carrying ClC-3 gene (pEZ-M03-ClC-3, GFP tagged) was successfully transfected into CNE-2Z cells, demonstrated by the fluorescent imaging analysis (Fig. 4A) and flow cytometry (Fig. 4B). The transfection efficiency obtained by analyzing the fluorescence intensity of individual cells using flow cytometry was 69.6 ± 2.9%. The expression level of ClC-3 proteins was increased by (65 ± 23)% 48 h after transfection (vs control, Fig. 4C,D).

**Figure 5:**
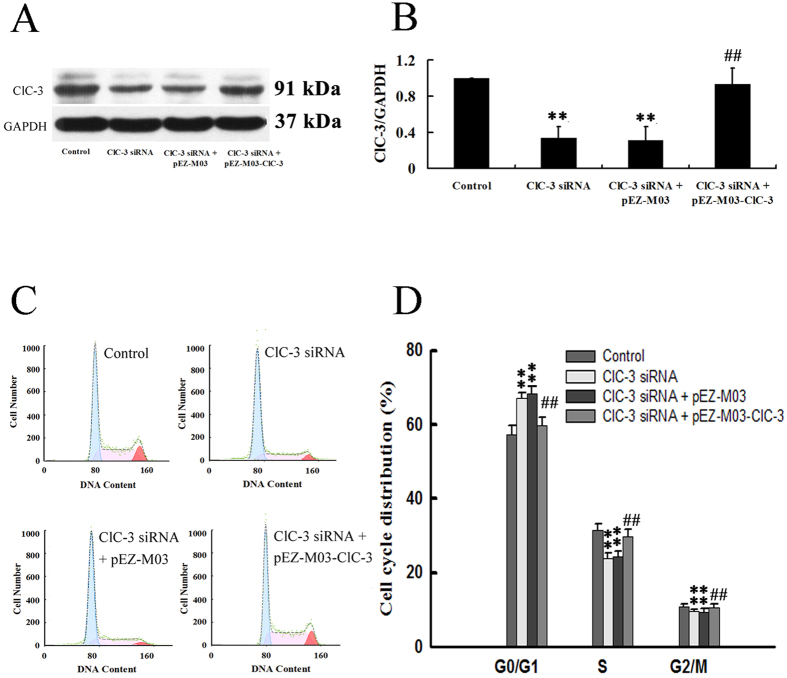
Rescue of CNE-2Z cells from ClC-3 siRNA-induced arrest of the cell cycle by reconstitution of ClC-3 expression. In the rescue experiment, cells were transfected with ClC-3 siRNA for 24 h, and then transfected with the ClC-3 plasmid (pEZ-M03-ClC-3) or the control plasmid (pEZ-M03) for 48 h. (**A**) Representative Western blot of ClC-3 and control GAPDH proteins in the control cells and the cells transfected with ClC-3 siRNA or ClC-3 siRNA + pEZ-M03 or pEZ-M03-ClC-3 (see rescue methods) for 48 h. (**B**) Quantitative analysis of Western blot (mean ± S.E., n = 3). (**C**) Cell cycle distribution detected by the flow cytometry. (**D**) Quantitative analysis of cell cycle distribution (mean ± S.E., n = 3). **P < 0.01 (vs. Control). ^##^*P* < 0.01 (vs. ClC-3 siRNA).

In rescue experiments, CNE-2Z cells were transfected with ClC-3 siRNA for 24 h, and then transfected with the ClC-3 plasmid (pEZ-M03-ClC-3) or the control plasmid (pEZ-M03) for 48 h. It was shown that ClC-3 expression, which had been knocked down by ClC-3 siRNA, could be recovered by pEZ-M03-ClC-3 transfection, demonstrated by the Western blotting analysis (Fig. 5A,B). The analysis of cell cycle distribution with flow cytometry indicated that reconstitution of ClC-3 expression by pEZ-M03-ClC-3 transfection released the CNE-2Z cells from the ClC-3 siRNA-induced arrest of the cell cycle (Fig. 5C,D). The G0/G1 population was decreased from (69.9 ± 3.0)% (ClC-3 siRNA only) to (59.8 ± 2.1)% (ClC-3 siRNA plus pEZ-M03-ClC-3), which was not significantly different from that in the control cells (56.8 ± 2.8)%. The results demonstrate that ClC-3 siRNA-induced arrest of the cell cycle can be rescued by ClC-3 reconstitution.

### ClC-3 siRNA reduced the expression of cyclin D1 in CNE-2Z cells

**Figure 6:**
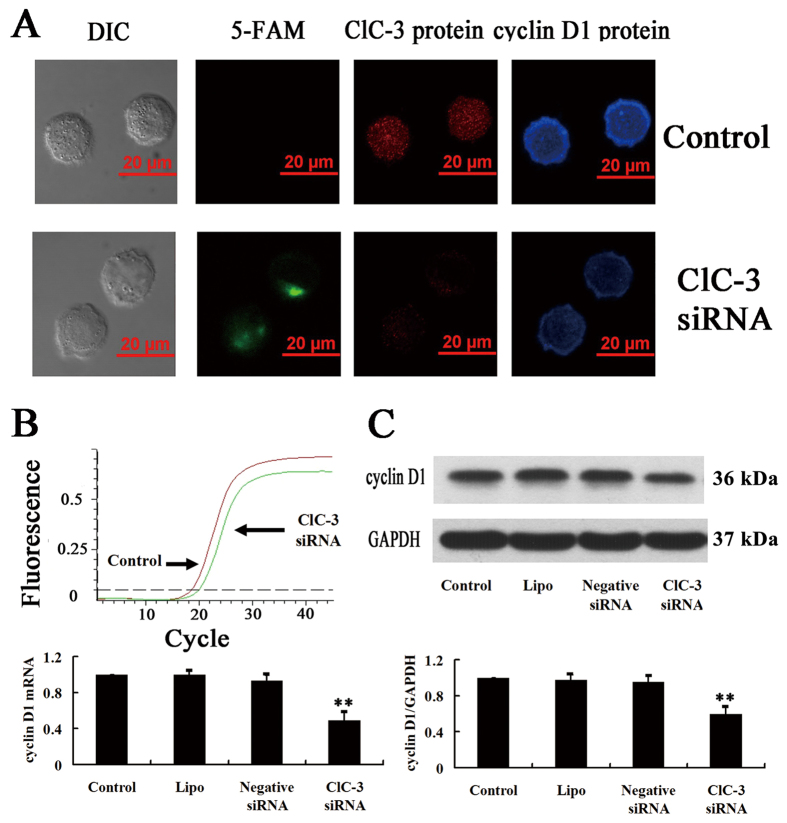
Inhibition of cyclin D1 expression by ClC-3 siRNA in CNE-2Z cells. (**A**) The transmitted light images obtained by the differential interference contrast (DIC) microscopy and the fluorescent images obtained by the confocal microscopy, showing the uptake of ClC-3 siRNA (labeled with 5-FAM, green), ClC-3 immuofluorescence (labeled with Cy3, red) and cyclin D1 immuofluorescence (labeled with Alexa Fluor ^®^ 405, blue) in the control and ClC-3 siRNA-treated groups. (**B**) Analysis of cyclin D1 mRNA expression in the control cells and the cells treated with the ClC-3 siRNA for 48 h. The upper panel shows the representative real-time PCR curves in the control and ClC-3 siRNA-treated groups. The lower panel shows the quantitative analysis of cyclin D1 mRNA expression. (**C**) Analysis of the expression of cyclin D1 proteins and the control GAPDH proteins. The upper and lower panels show the representative blot and the quantitative analysis, respectively. Data in B and C are mean ± S.E. (n = 3). **P < 0.01 (vs. Control).

The above results demonstrate that the chloride channel protein ClC-3 is involved in the regulation of cell cycle progression from the G1 phase to the S phase. The effect of knockdown of ClC-3 expression by ClC-3 siRNA on the expression of cyclin D1, a key protein that stimulates exit from G0 and regulates progression through the restriction point at G1, was then investigated. The results indicated that the expression of the cyclin D1 mRNA and protein was down-regulated when the expression of ClC-3 was knocked down by the ClC-3 siRNA (Fig. 6).

In immunofluorescence experiments, the second antibody for detecting ClC-3 was labeled with Cy3 (red fluorescence), and the second antibody for detecting cyclin D1 was conjugated with the fluorophore 405 (blue fluorescence). As shown in the upper panel of Fig. 6A, the control nasopharyngeal carcinoma CNE-2Z cells expressed ClC-3 (red) and cyclin D1 (blue). In the cells transfected with ClC-3 siRNA (labeled with fluorophore 5-FAM, green) for 48 h, both the red and blue fluorescence was significantly weaker than that in the control cells, indicating that the expression of both ClC-3 and cyclin D1 was down-regulated by the ClC-3 siRNA treatment (lower panel in Fig. 6A). The expression of cyclin D1 mRNA detected by the real-time RT-PCR was also down-regulated by (50.9 ± 8.6)% (vs control) after treatment with ClC-3 siRNA (Fig. 6B). To quantify the effect of knockdown of ClC-3 expression by ClC-3 siRNA on the expression of cyclin D1, the Western blotting technique was used to detect expression levels of cyclin D1 proteins. The results showed that the treatment of cells with 100 nM ClC-3 siRNA for 48 h decreased the expression of cyclin D1 proteins. The expression level of cyclin D1 proteins was decreased by (40.3 ± 2.0)% by the ClC-3 siRNA treatment (vs control, Fig. 6C).

In the cells treated with 100 nM control siRNA plus the transfection agent lipofectamine^TM^ 2000 or with lipofectamine^TM^ 2000 alone for 48 h, the expression of cyclin D1 mRNA and proteins was not significantly different from that in the control cells (n = 3, *P* > 0.05, Fig. 6B,C). These results demonstrate that the expression of cyclin D1 was reduced after the expression of ClC-3 had been knocked down by the specific ClC-3 siRNA, suggesting that expression of cyclin D1 is regulated by ClC-3.

### Knockdown of intrinsic ClC-3 expression down-regulated CDK4/CDK6 expression and up-regulated p21/p27 expression

**Figure 7:**
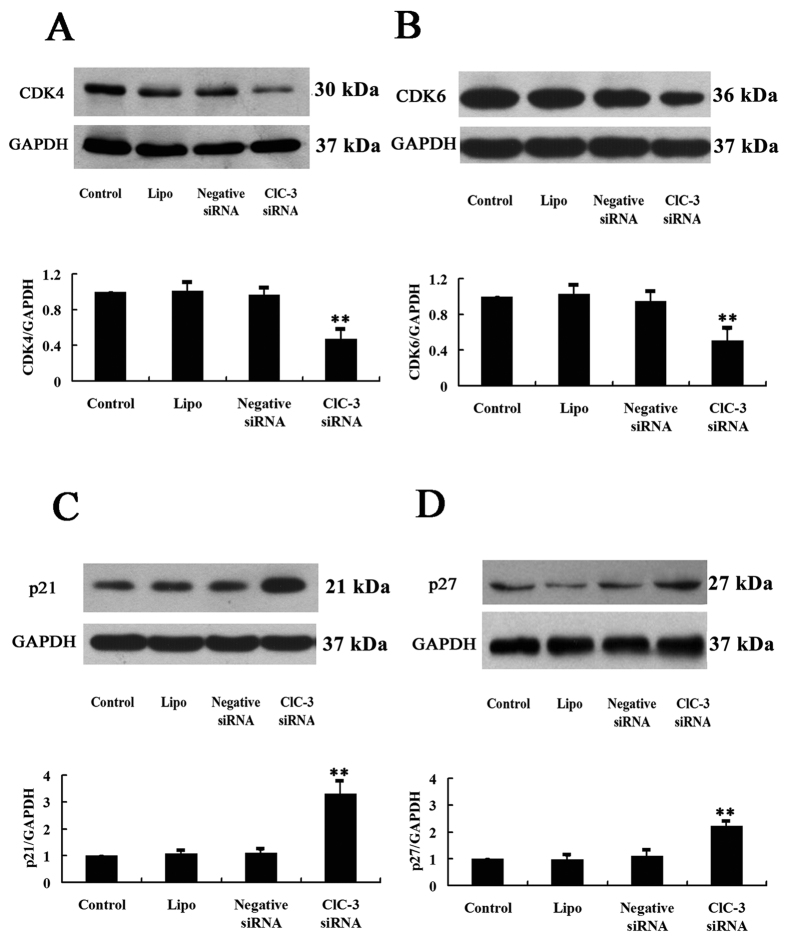
Inhibition of the expression of CDK4/CDK6 and enhancement of the expression of p21/p27 by ClC-3 siRNA in CNE-2Z cells. (**A–D**) show the analysis of the expression of CDK4, CDK6, p21 and p27 proteins by Western blot (with the reference protein GAPDH), respectively. The upper and the lower panels in each figure show the representative Western blot and the quantitative analysis of protein expression, respectively, in the control cells and the cells treated with the transfection reagent lipofectamine 2000 alone (Lipo), or with 100 nM negative siRNA or ClC-3 siRNA plus lipofectamine 2000 for 48 h (mean ± S.E., n = 3). **P < 0.01 (vs. Control).

Cyclin-dependent kinases (CDKs) are a family of kinases first discovered for their role in regulating the cell cycle. CDK4 and CDK6 are two key partners of cyclin D1 in regulation of the progress of the cell cycle in the G1 phase. Western blot results showed that nasopharyngeal carcinoma CNE-2Z cells expressed CDK4 and CDK6 (Fig. 7A,B). The expression of CDK4 and CDK6 was reduced by (52.5 ± 4.5)% (Fig. 7A) and (49.3 ± 7.5)% (Fig. 7B) respectively in the cells treated with ClC-3 siRNA, compared with the control group. In the cells treated with the transfection reagent lipofectamine^TM^ 2000 alone or with the negative control siRNA plus lipofectamine^TM^ 2000, the expression of CDK4 and CDK6 was not significantly changed.

Cyclin-dependent kinase inhibitors (CKIs) are the proteins that inhibit the activities of cyclin-dependent kinases. Cell cycle progression is negatively controlled by cyclin-dependent kinase inhibitors. To investigate further the action mechanism of the ClC-3 on cyclin D1, we used the Western blot technique to detect the changes of the expression of the cyclin-dependent kinase inhibitors p21 and p27. Results showed that p21 expression was increased by (231.3 ± 44.2)% (Fig. 7C) and p27 expression was increased by (120.6 ± 17.1)% (Fig. 7D) in the cells treated with ClC-3 siRNA, compared with those in the control group. In the cells treated with lipofectamine^TM^ 2000 alone or with negative siRNA plus lipofectamine^TM^ 2000, neither the expression of p21 nor that of p27 was significantly changed (Fig. 7C,D).

### p21/p27 is necessary for the regulatory function of ClC-3 on CDK4/CDK6 expression

To study further the relationships between ClC-3, p21/p27 and CDK4/CDK6, the expression of p21/p27 was first knock-downed by p21 and p27 siRNAs, and the regulatory action of ClC-3 on CDK4/CDK6 expression was then investigated. Cells were treated with p21 siRNA and p27 siRNA for 24 h, and then transfected with ClC-3 siRNA for another 48 h before Western blot analysis.

**Figure 8:**
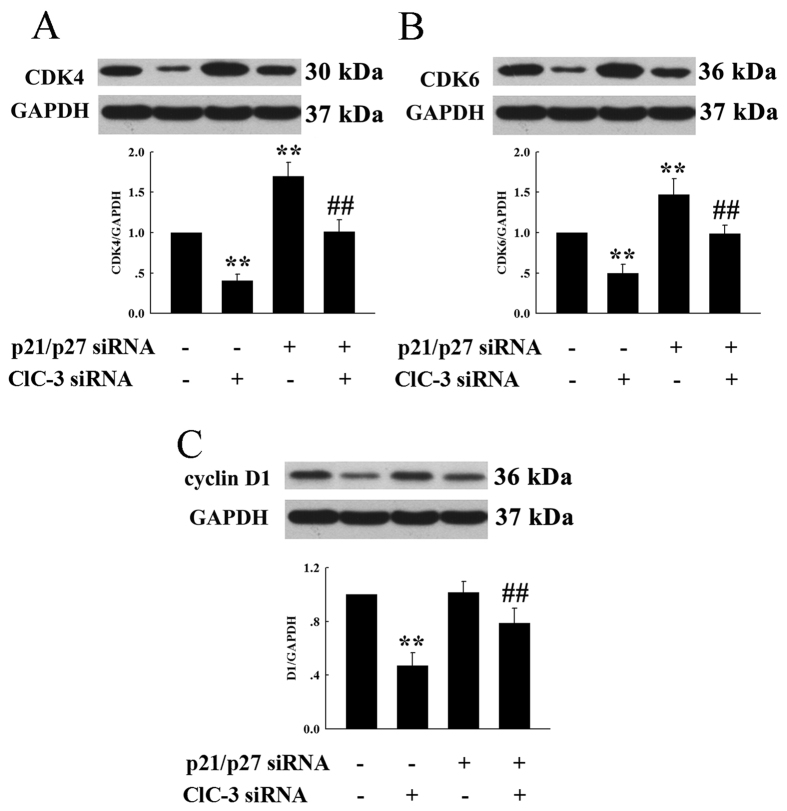
Effects of ClC-3, p21 and p27 siRNAs on the expression of CDK4, CDK6 and cyclin D1. The cells were incubated in the control medium or treated with 100 nM specifically-targeting siRNA for 48 h. In the combination group, cells were pretreated with the p21 and p27 siRNAs for 24 h, and then transfected with ClC-3 siRNA for 48 h. Analysis of the expression of CDK4, CDK6 and cyclin D1 proteins and the control GAPDH proteins by Western blot in different treatments is shown in (**A–C**), respectively. The upper and the lower panels in each figure show the representative blotting and the quantitative analysis, respectively (mean ± S.E., n = 3). **P < 0.01 (vs. Control). ^##^*P* < 0.01 (vs. ClC-3 siRNA).

As shown in Fig. 8A,B, knockdown of ClC-3 expression by ClC-3 siRNA could down-regulate CDK4/CDK6 expression. However, the pretreatment of cells with p21/p27 siRNA depleted the effects of ClC-3 siRNA on CDK4/CDK6 expression, indicating that p21/p27 is necessary for ClC-3 siRNA-induced down-regulation of CDK4/CDK6 expression. It was also demonstrated in this study that the knockdown of p21/p27 expression by p21/p27 siRNA significantly increased CDK4/CDK6 expression. These results suggest that ClC-3 may positively regulate CDK4/CDK6 expression by suppression of p21 and p27 expression.

The expression of cyclin D1 was also decreased by the transfection with ClC-3 siRNA alone (Fig. 8C), but was not significantly changed by p21/p27 siRNAs. Furthermore, pretreatment of cells with p21/p27 siRNA did not significantly interfere with the effects of ClC-3 siRNA on cyclin D1 expression. The results indicate that p21/p27 is not required for the regulation of cyclin D1 expression by ClC-3.

### p21/p27 is necessary for the ClC-3 siRNA-induced arrest of CNE-2Z cells at the G0/G1 phase

**Figure 9:**
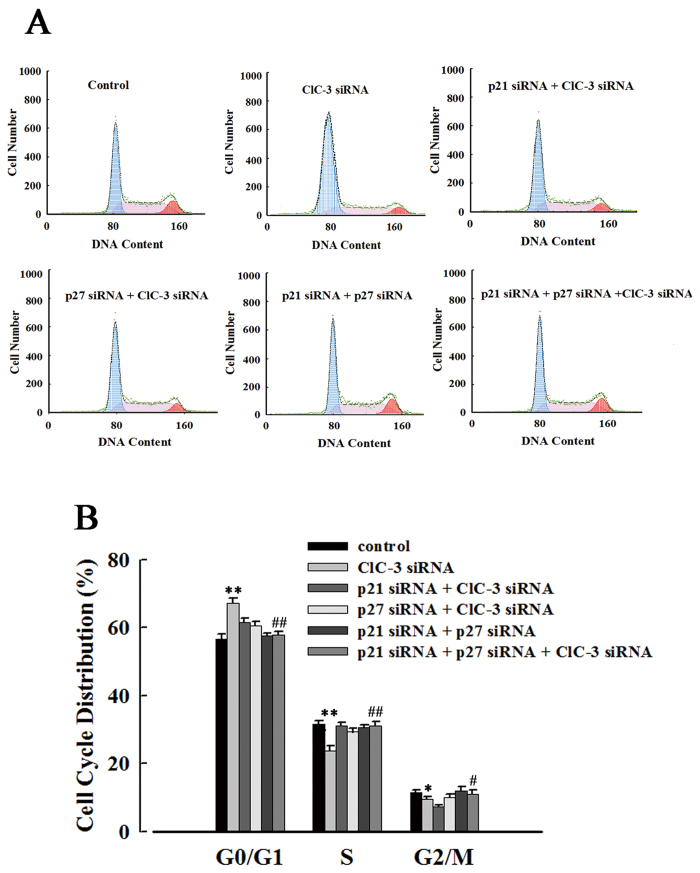
Prevention of ClC-3 siRNA-induced arrest of CNE-2Z cells at the G0/G1 phase by p21 siRNA and p27 siRNA treatments. (**A**) Cell cycle distribution detected by flow cytometry in the control cells and the cells treated with lipofectamine 2000 alone (Lipo), or with 100 nM negative siRNA or specifically-targeting siRNA plus lipofectamine 2000 for 48 h. (**B**) Quantitative analysis of cell cycle distribution (mean ± S.E. of three experiments). *P < 0.05; **P < 0.01 (vs. Control). ^##^*P* < 0.01 (vs. ClC-3 siRNA).

Cells were treated with p21 siRNA and p27 siRNA for 24 h, and then were transfected with ClC-3 siRNA for another 48 h before cell cycle analysis. Flow cytometry was used to detect the cell cycle distribution in different groups of cells (Fig. 9A). It was shown that ClC-3 siRNA inhibited the cell cycle progress. In the cells transfected with ClC-3 siRNA alone, the percentage of cells in the G0/G1 phase was increased to (69.9 ± 3.0)% from (56.8 ± 2.8)% (control), that in the S phase was decreased from (32.1 ± 1.7)% (control) to (23.5 ± 1.5)%, and that in the G2/M phase was reduced from (11.0 ± 1.1)% (control) to (6.5 ± 1.5)% (Fig. 9B).

Further experiments indicated that the ClC-3 siRNA-induced cell arrest at the G0/G1 phase was prevented by the pretreatments of cells with p21 and p27 siRNAs. In the cells transfected first with p21/p27 siRNAs and then with ClC-3 siRNAs, the percentages of cells in G0/G1, S and G2/M phases were (57.9 ± 1.9)%, (31.1 ± 1.3)% and (11.0 ± 1.1)% (Fig. 9B), which were similar to those of the control cells, but were significantly different from those of the group transfected with ClC-3 siRNA alone. The results suggest that ClC-3 siRNA may interfere with the cycle distribution by regulating the expression of p21 and p27.

### Knockdown of intrinsic ClC-3 expression arrested cells at the G0/G1 phase, down-regulated cyclin D1 and CDK4/CDK6 expression and up-regulated p21/p27 expression in HeLa cells

The effects of knockdown of ClC-3 expression by ClC-3 siRNA on cell cycle progression and expression of cyclin D1, CDK4, CDK6, p21 and p27 were also tested in the HeLa cell, a cell line derived from cervical cancer cells. It was found in HeLa cells that ClC-3 siRNA could inhibit the progression of cells into the S phase from the G1 phase, suppress the expression of cyclin D1, CDK4 and CDK6 and up-regulate the expression of p21 and p27 (data not shown).

## Discussion

Cell cycle regulation is a complex and precise physiological process. It is well known that cyclins-CDKs-CDKIs play a central role in regulation of the cell cycle. Recently, ion channels have been suggested to be involved in the control of cell cycle progress^4^,^5^,^11^,^17^,^18^. In this study, it was demonstrated that the ClC-3 chloride channel protein could regulate the cell cycle by modulating the expression of cyclins-CDKs-CDKIs.

Firstly, we demonstrated that ClC-3 was a key molecule for activation of volume-sensitive chloride currents and the cell swelling-induced regulatory volume decrease process in nasopharyngeal carcinoma CNE-2Z cells. The siRNA technology was used to silence the expression of ClC-3 specifically. It was shown that the ClC-3 siRNA used in this study was highly efficient in knocking down the expression of ClC-3. Knockdown of ClC-3 expression prevented the activation of the hypotonicity-induced chloride current and regulatory volume decrease. These data support the hypothesis that ClC-3 is an important component and/or regulator of the volume-activated chloride channel, but do not exclude the possibility that ClC-3 regulates the volume-activated chloride channel indirectly by activating or inducing production of other regulators. It was reported that activation of the swelling-activated chloride current by tumor necrosis factor-alpha (TNF-alpha) requires ClC-3- dependent endosomal reactive oxygen production^25^. Recent evidence suggests that ClC-3 can work as a Cl^−^/H^+^ antiporter^26^,^27^.

It was previously proved by us that the expression of volume-activated chloride currents and the capacity of regulatory volume decrease were cell cycle dependent; pharmacological blockage of chloride channels attenuated the capacity of cell volume regulation, and arrested CNE-2Z cells in the G0/G1 phase^1^,^5^. Here we showed that ClC-3 was the main molecule associated with the activation of volume-activated chloride current and regulatory volume decrease, thus, silencing the expression of ClC-3 should interfere with the progress of the cell cycle. This was proved by our next experiments. It was found that knockdown of ClC-3 expression arrested CNE-2Z cells at the G0/G1 phase, indicating that ClC-3 plays important roles in regulation of the cell cycle in CNE-2Z cells. The association of ClC-3 with cell cycle progress is further confirmed by our rescue experiments, in which reconstitution of ClC-3 expression released the cells from the G0/G1 arrest caused by knocking down ClC-3 expression with the ClC-3 siRNA.

How does ClC-3 regulate the cell cycle? It is known that cell cycle progression is governed by the well-orchestrated activation and inactivation of CDKs^28^,^29^. G1 to S phase transition plays a crucial role in maintaining the genomic integrity because this phase is critically linked to external stimuli and also commits the cells to DNA replication and subsequent mitosis^30^,^31^. G1 checkpoint abrogation is a common phenomenon in carcinogenesis which endows the tumor cells with limitless replicative potential^32^. Cyclin D1 is a key factor in the regulation of cells to pass through the G1 restriction point, and was confirmed to have close contact with a variety of proteins^20^,^22^,^28^. Many oncogenes, tumor suppressor genes and apoptosis-related genes are closely related to cyclin D1^33^,^34^,^35^,^36^,^37^,^38^,^39^,^40^. Cyclin D1 is synthesized in the G1 phase, and then forms the cyclin Dl-CDK4 and/or cyclin Dl-CDK6 complexes, resulting in the activation of CDK4/6. The activated CDK4/6 promotes DNA transcription, regulates DNA replication and repair, and facilitates cells to pass through the G1 restriction point. All these data indicate that the cyclin D1-CDK4/6 complex is the key determinant that promotes the cell cycle progress from the G1 phase to the S phase. ClC-3 may control the cell cycle progress by regulating the expression of cyclin D1 and/or CDK4/6. This is supported by our results. It was found that knockdown of the expression of ClC-3 by specific ClC-3 siRNA down-regulated the expression of cyclin D1, CDK4 and CDK6. These results indicate that cyclin D1 and CDK4/6 can be the downstream targets of ClC-3. ClC-3 can regulate cell cycle progress by up-regulating the expression of cyclin D1 and CDK4/6.

ClC-3 may regulate the expression of cyclin D1 and CDK4/6 directly or indirectly. The activities of the cyclin/CDK complexes can be inhibited by cyclin-dependent kinase inhibitors (CDKI). Activation of CDKIs can prevent the appearance of the disorder in the cell cycle machinery. p21 and p27 can bind to cyclin/CDK complexes. p21 and p27 physically interact with CDK via their amino terminal domain and inhibit CDK kinase activity. The binding of p21 and p27 to the cyclin/CDK complexes results in inhibition of the complex activities and the arrest of the cell cycle. The expression of cyclins and CDKs can also be affected by p21 and p27. Activating the G1 checkpoint by up-regulating the expression of p21 and p27 is thus a logical approach for controlling cancer cell proliferation. There is a possibility that ClC-3 may indirectly regulate the activities and/or expression of cyclin D1-CDK4/6 by modulating the expression of p21 and p27. This postulation is supported by our results that the expression of p21 and p27 was indeed up-regulated after the knockdown of ClC-3 expression by the ClC-3 siRNA. This result suggests that p21 and p27 are negatively regulated by ClC-3; ClC-3 can indirectly increase the expression of cyclin D1-CDK4/6 complexes by suppressing the expression of p21 and p27.

The postulation of indirect regulation of cyclin D1-CDK4/6 expression by ClC-3 through p21 and p27 is supported by our further experiments. It was found by us that ClC-3 siRNA suppressed the expression of CDK4 and CDK6; the pretreatments of cells with p21 and p27 siRNAs prevented the inhibitory effect of ClC-3 siRNA on the expression of CDK4 and CDK6. These results suggest that the expression of p21 and p27 is required for the ClC-3 siRNA action on CDK4 and CDK6 expression; ClC-3 may down-regulate p21 and p27 expression, and then release the inhibitory action of p21 and p27 on the expression of CDK4 and CDK6, resulting in the increase of CDK4 and CDK6 expression. Our experiments also showed that ClC-3 siRNA could inhibit the expression of cyclin D1. Pretreatments of cells with p21 and p27 siRNA did not significantly change the inhibitory effect of ClC-3 siRNA on cyclin D1 expression. This result suggests that p21 and p27 are not required for ClC-3 siRNA action on cyclin D1 expression. ClC-3 may promote the expression of cyclin D1 in a p21/p27-independent manner.

ClC-3 may promote the cell cycle progress from the G1 phase to the S phase by up-regulation of cyclin D1-CDK4/6 expression directly, or indirectly via down-regulation of the CDKIs, p21 and p27. In our experiments, it was shown that ClC-3 siRNA increased the percentage of cells in the G0/G phase, but p21 siRNA and/or p27 siRNA treatments depleted the ClC-3 siRNA effect on the cell cycle distribution. These results indicate that p21 and p27 are required for the ClC-3 siRNA action on the cell cycle. The p21 and p27 pathways are the important pathways for the regulation of the cell cycle by ClC-3. ClC-3 siRNA may interfere with the cell cycle progress by the following pathway: ClC-3 siRNA suppresses ClC-3 expression; down-regulation of ClC-3 expression releases the expression of p21 and p27 from the inhibitory effect of ClC-3, resulting in the increase of p21 and p27 expression; the increased p21 and p27 down-regulate and/or inhibit the expression and activities of cyclin D1-CDK4/6 complexes, and arrest cells in the G0/G1 phase. The results suggest that ClC-3 can promote the cell cycle progress by suppressing p21 and p27 expression. In this study, it was also shown that the cell cycle distribution was not significantly changed by p21 and p27 siRNA in nasopharyngeal carcinoma CNE-2Z cells. Similar effects has also been shown in human prostate cancer DU145 cells^41^. When both p21 and p27 siRNAs were used together to create a double knockdown condition, there was no change in cell cycle distribution even though protein expression of both the molecules was ablated. These results imply that the expression of p21 and p27 is at a relative low level in the control condition, p21 and p27 in this level do not significantly affect cell cycle progress. Once the suppression from ClC-3 has been eliminated by ClC-3 siRNA treatment, the expression of p21 and p27 would be increased, which then suppress the cell cycle progress.

We have also investigated the role of ClC-3 in the cell cycle by using another cell line (the HeLa cell). Similar to the effect in the CNE-2 cell, knockdown of ClC-3 expression by ClC-3 siRNA down-regulated cyclin D1 and CDK4/CDK6 expression, up-regulated p21/p27 expression and arrested cells at the G0/G1 phase in HeLa cells. The data imply that the ClC-3 siRNA-induced cell cycle changes may be a more common phenomenon.

As discussed above, ClC-3 is suggested to be a component and/or a regulator of the volume-activated Cl^−^ channel in the plasma membrane, and to be involved in regulation of the cell cycle by way of suppressing p21 and p27 expression and promoting cyclin D1 and CDK4/6 expression in CNE-2Z cells. How does the ClC-3 protein function in regulation of the expression of these genes? Our previous work demonstrates that ClC-3 is dynamically translocated between plasma membrane- cytoplasm-nucleus in the cell cycle, and is predominantly located in the nucleus in the G1 phase^21^. It is possible that the nucleus-translocated ClC-3 directly exerts its nuclear effects on gene expression, or indirectly modulates gene expression by activation of other transcription factors. There is evidence that ClC-3 is necessary for the activation of mitogenic events induced by TNF-alpha^42^, and TNF-alpha can exert a mitogenic effect by activating the transcription factor NF-kappa B and induce cyclin D1 expression^43^. ClC-3 is also required for cytokine-induced activation of NF-kappa B and production of reactive oxygen species (ROS), which are important signaling molecules for many physiological and pathological activities including cell proliferation^44^,^45^,^46^. The production of ROS and activation of NF-kappa B may thus be important events linking ClC-3 to the cell cycle.

In conclusion, we have demonstrated that ClC-3 plays an important role in regulation of CNE-2Z cells to pass through the G1-S transition point of the cell cycle. ClC-3 may regulate cell cycle progression by up-regulating the expression and activities of cyclin D1-CDK4/6 complexes through suppression of p21 and p27 expression in nasopharyngeal carcinoma CNE-2Z cells. ClC-3 may be an important target for cancer therapy.

## Methods

### Cell culture

CNE-2Z (the poorly differentiated human nasopharyngeal carcinoma cell line) was kindly provided by Professor Weiping Tang (Department of Pathology, Guangdong Medical College, China). ClC-3 chloride channel proteins were overexpressed in CNE-2Z cells. The CNE-2Z cell and the Hela cell (derived from cervical cancer cells) were routinely cultured in the RPMI 1640 medium (GIBCO) with 10% newborn calf serum, 100 IU/ml penicillin and 100 μg/ml streptomycin in a humidified atmosphere of 5% CO_2_ and 95% air at 37 °C. The cells were subcultured every 48 h.

### Transfection of CNE-2Z cells with ClC-3, p21 and/or p27 siRNA

The siRNA against human ClC-3, p21 and p27 genes were labelled with 5-FAM, and they were synthesized by Shanghai GenePharma Company (Shanghai, China).

The sequence of the ClC-3 siRNA was 5′-CAAUGGAUUUCCUGUCAUATT-3′, its complementary strand was 5′-UUCUCCGAACGUGUCACGUTT-3′. The sequence of the p21 siRNA was 5′-GAUGGAACUUCGACUUUGUTT-3′, its complementary strand was 5′-ACAAAGUCGAAGUUCCAUCTT-3′. The sequence of the p27 siRNA was 5′-GCAACCGACGAUUCUUCUATT-3′, its complementary strand was 5′-UAGAAGAAUCGUCGGUUGCTT-3′. The sequence of negative control siRNA was 5′-UUCUCCGAACGUGUCACGUTT-3′, its complementary strand was 5′-ACGUGACACGUUCGGAGAATT-3′.

The siRNAs were transfected with Lipofectamine^TM^ 2000 (Invitrogen, Carlsbad, USA) in the final concentration of 100 nM, following the manufacturer’s instruction. The cells were further incubated in the normal growth condition for 48 h or more before experiments.

### Construction and transfection of ClC-3 expression plasmids

The full-length of human ClC-3 cDNA was inserted into the eukaryotic expression vector (pEZ-M03) carrying the gene of green fluorescent protein (GFP). The constructed plasmid (pEZ-M03-ClC-3) and the control plasmid (pEZ-M03) were transfected into CNE-2Z cells in the presence of the transfection reagent Lipofectamine^TM^ 2000 following the manufacturer’s instruction. The cells were then cultured in the normal growth condition for 48 h. 2 μg of plasmids and 5 μl of Lipofectamine ^TM^ 2000 were used for each well of the six-well cell culture plate.

In the experiments rescuing the cells from ClC-3 siRNA-induced inhibition, cells were transfected with ClC-3 siRNA in the presence of Lipofectamine 2000 and incubated in the normal growth medium for 24 h. The cells were then transfected with plasmids (pEZ-M03-ClC-3 or pEZ-M03) with the help of Lipofectamine 2000, and cultured in the normal growth condition for 48 h.

### Detection of siRNA and plasmid transfection efficiency

The transfection efficiency of siRNAs and plasmids were detected by the fluorescent microscopy and flow cytometry. After transfection with siRNA (ClC-3, p21 and/or p27 siRNA) or plasmids (pEZ-M03 or pEZ-M03-ClC-3) for about 6–24 h, the cells were washed with PBS 3 times. Cell images were then taken under a confocal microscope (C1-si, Nikon instrumetns, Japan). The fluorescence of the control and the transfected cells was further analyzed by a flow cytometer (EPTCSXL-31240, Coulter, USA), using an excitation wavelength of 488 nm. The transfection efficiency was obtained by analyzing the fluorescence intensity of individual cells using flow cytometry.

### Whole cell current recordings and solutions.

The osmolarity of the isotonic perfusion solution was 300 mOsmol/L, and it contained (mM): 70 NaCl, 0.5 MgCl_2_, 2 CaCl_2_, 10 HEPES and 140 D-mannitol. The osmolarity of the hypotonic perfusion solution was 160 mOsmol/L (47% hypotonic, compared to that of the isotonic solution), and it contained (mM): 70 NaCl, 0.5 MgCl_2_, 2 CaCl_2_ and 10 HEPES. The pipette solution contained (mM) 70 N-methyl-D-glucamine chloride (NMDG-Cl), 1.2 MgCl_2_, 10 HEPES, 1 EGTA, 140 D-mannitol and 2 ATP, and its osmolarity was adjusted to 300 mOsmol/L. The pH of perfusion and pipette solutions were adjusted to 7.4 and 7.25, respectively.

Whole cell currents were recorded with the EPC-7 patch clamp amplifier (HEKA, Germany). The membrane potential was held at the Cl^−^ equilibrium potential (0 mV) and stepped to the 200 ms-pulses of ±80, ±40 and 0 mV in sequence and repeatedly, with 4 s intervals between pulses. Voltages and currents were recorded by using a laboratory interface (CED 1401, Cambridge, UK). Currents were measured at 10 ms after onset of voltage steps.

### Cell volume measurements

The cells were digested with 0.25% trypsin, dropped on small glass coverslips and incubated for 1 h to achieve cell adhesion. The coverslip with cells was placed in a special perfusion bath. The cells were perfused in sequence with the isotonic perfusion solution for 5 min, hypotonic perfusion solution for 25 min and isotonic perfusion solution for 5 min. Cell images were captured every 1 minute by a CCD camera (Mono CCD625, Leica, Germany) and analyzed with the Scion software (Scion Corporation, USA). Cell volume was computed from cell diameters.

Cell volume was normalized with the formula: V_st_ = V_t_ ÷ V_0_ × 100% (V_st_, standardized cell volume; V_t_, the real-time measured cell volume; V_0_, the cell volume before hypotonic stimulation). The hypotonicity-induced regulatory volume decrease (RVD) was calculated with the following formula: RVD (%) = (V_max_−V_min_) ÷ (V_max_−V_ctrl_) × 100% (V_ctrl_, the cell volume in isotonic perfusion solution; V_max_, the maximum expansion volume in the hypotonic perfusion solution; V_min_, the volume before re-perfused with the isotonic perfusion solution). All experiments were carried out at room temperature (20~24 °C).

### Cell cycle analysis by flow cytometry

The flow cytometry was used to analyze the effects of ClC-3, p21 and/or p27 siRNAs on the cell cycle distribution of CNE-2Z cells. CNE-2Z cells were digested with 0.25% trypsin 48 h after transfected with siRNAs and collected by centrifugation at 200 g for 5 min at 4 °C. The cells were washed with ice-cold phosphate-buffered saline (PBS) 3 times and fixed in 70% ethanol for 24 h. The samples were stained with PBS containing 50 μg/mL of propidium iodide, 10 μg/mL RNase A, 0.1% sodium citrate and 0.1% Triton X-100. At last, the cell cycle distribution was detected by the flow cytometer (EPTCSXL-31240, Coulter, USA). The wavelength used to determinate PI/DNA is 488 nm.

### Immunofluorescence analysis of ClC-3 and cyclin D1

CNE-2Z cells cultured on glass coverslips were fixed in paraformaldehyde (4%) for 30 min at room temperature, and then permeabilized with Triton X-100 (0.5%, v/v in PBS) at 4 °C for 5 min. After blocking with 5% normal goat serum for 60 min at room temperature, the cells were then incubated with the ClC-3 primary antibody (1:100, Abcam, USA) and cyclin D1 primary antibody (1:100, Cell Signalling Technology, USA) at 4 °C overnight. The next day, the coverslips were rinsed for 5 min with PBS three times and incubated with the secondary antibodies (1:100), Alexa Fluor® 405 (blue)-labeled goat anti-rabbit IgG and Cy3 (red)-conjugated goat anti-mouse IgG (Invitrogen, USA), in dark for 30 min at 37 °C. Between incubation steps, cells were rinsed with PBS. Cells were observed by the Nikon C1-si laser-scanning confocal microscope (Nikon instrumetns, Japan).

### Real-time PCR analysis for ClC-3 and cyclin D1

Total RNA was isolated from CNE-2Z cells using Trizol reagent (TaKaRa, Kyoto, Japan) and was reverse transcribed using the PrimeScript® RT reagent kit (TaKaRa, Kyoto, Japan) following the instructions provided by the manufacturer. The real-time PCR was performed using the SYBR® PrimeScript™ RT-PCR Kit II (TaKaRa, Kyoto, Japan), and the reactions were carried out in the BioRad real-time PCR system (BioRad, USA). The nucleotide sequences of primers were as follows:

5′-TTGCCTACTATCACCACGAC-3′ (forward) and 5′-GCATCTCCAACCCATTTACT-3′ (reverse) for ClC-3, 5′-GTGCATCTACACCGACAACTCCA-3′ (forward) and 5′-TGAGCTTGTTCACCAGGAGCA-3′ (reverse) for cyclin D1, 5′-GGTGGTCTCCTCTGACTTCAACA-3′ (forward) and 5′-GTTGCTGTAGCCAAATTCGTTGT-3′ (reverse) for GAPDH.

The PCR reaction was performed as follows: 95 °C for 30 seconds, and then 95 °C for 5 seconds annealing at 60 °C for 20 seconds (40 cycles). The ClC-3 and cyclin D1 gene signals were normalized to GAPDH, and the data were calculated by using the formula 2^−ΔΔCt^.

### Western blot analysis

CNE-2Z cells were rinsed with ice-cold PBS 3 times and bathed in RIPA lysis buffer with 1% PMSF for 30 min. Proteins were separated with 10% (for GAPDH, ClC-3, cyclin D1, CDK4 and CDK6) or 12% SDS-PAGE (for p21 and p27), and transferred to nitrocellulose membranes. Membranes were blocked at room temperature for 1.5 h, incubated with primary antibodies at 4 °C overnight, rinsed with TBST for 10 min 3 times, and then incubated with the HRP-linked secondary antibody at room temperature for 1 h. ECL chemiluminescence system was used and the blots were exposed to X-ray films, which were then detected by the gel analysis system. The primary antibodies for cyclin D1, CDK4, CDK6, p21, p27 and GAPDH were purchased from Cell Signalling Technology (USA) and diluted 1:1000. The primary antibody, rabbit polyclonal anti-ClC-3 (ab86192, Abcam, USA) was diluted 1:1000. The sizes of cyclin D1, CDK4, CDK6, p21, p27, ClC-3 and GAPDH bands are 36, 30, 36, 21, 27, 91 and 37 kDa, respectively. GAPDH was chosen as the reference because GAPDH is a house keeping gene and expressed steadily in many kinds of cells including nasopharyngeal carcinoma cells. The bands were quantified by optical density ratios to GAPDH using a BI2000 image analysis system (Chengdu TME Technology Co Ltd, Chengdu, China).

### Statistical Analysis

All the experiments were repeated at least three times. Data were analyzed using the statistical software SPSS 13.0 (Chicago, USA) and expressed as mean ± standard error (S.E.). The significance of differences was determined using the ANOVA test for multiple group comparisons. Statistical significance was accepted at *P* < 0.05.

## Additional Information

**How to cite this article**: Ye, D. *et al*. ClC-3 Chloride Channel Proteins Regulate the Cell Cycle by Up-regulating cyclin D1-CDK4/6 through Suppressing p21/p27 Expression in Nasopharyngeal Carcinoma Cells. *Sci. Rep.* **6**, 30276; doi: 10.1038/srep30276 (2016).
